# Predicting the risk of pulmonary tuberculosis based on the neutrophil-to-lymphocyte ratio at TB screening in HIV-infected individuals

**DOI:** 10.1186/s12879-019-4292-9

**Published:** 2019-07-29

**Authors:** Reiko Miyahara, Surachai Piyaworawong, Vivek Naranbhai, Prarit Prachamat, Prapimporn Kriengwatanapong, Naho Tsuchiya, Jiraporn Wongyai, Surasit Bupachat, Norio Yamada, Surin Summanapan, Surakameth Mahasirimongkol, Hideki Yanai

**Affiliations:** 1 Department of Human Genetics, Graduate School of Medicine, The University of Tokyo, Tokyo, Japan; 2 Genome Medical Science Project, National Center for Global Health and Medicine, 1-21-1 Toyama, Shinjuku-ku, Tokyo, 162-8655 Japan; 3 Mae Chan Hospital, Chiang Rai, Thailand; 4 Massachusetts General Hospital, Boston, USA; 5 Centre for the AIDS Programme of Research in South Africa, Durban, South Africa; 6 Department of Preventive Medicine and Epidemiology, Tohoku Medical Megabank Organization, Tohoku University, Sendai, Japan; 7 TB/HIV Research Foundation, Chiang Rai, Thailand; 8 Research Institute of Tuberculosis (RIT), Anti-Tuberculosis Association (JATA), Tokyo, Japan; 9 Ministry of Public Health, Nonthaburi, Thailand; 10 JATA, Fukujuji Hospital, Tokyo, Japan

**Keywords:** Tuberculosis, TB screening, Neutrophil, Lymphocyte, HIV, Mortality

## Abstract

**Background:**

The neutrophil to lymphocyte ratio (NL ratio) has been reported to be a predictive biomarker of tuberculosis (TB). We assessed the association between the NL ratio and the incidence of active TB cases within 1 year after TB screening among HIV-infected individuals in Thailand.

**Methods:**

A day care center that supports HIV-infected individuals in northernmost Thailand performed TB screening and follow-up visits. We compared the baseline characteristics between the TB screening positive group and the TB screening negative group. The threshold value of NL ratio was determined by cubic-spline curves and NL ratios were categorized as high or low NL ratio. We assessed the association between NL ratio and progression to active TB within 1-year using the Cox-proportional hazard model.

**Results:**

Of the 1064 HIV-infected individuals who screened negative for TB at baseline, 5.6% (*N* = 60) eventually developed TB and 26 died after TB diagnosis. A high NL ratio was associated with a higher risk of TB (adjusted hazard ratio (aHR) 2.19, 95% CI: 1.23–3.90), after adjusting for age, sex, ethnicity, CD4 counts, and other risk factors. A high NL ratio in HIV-infected individuals with normal chest X-ray predicted TB development risk. In particular, a high NL ratio with TB symptoms could predict the highest risk of TB development (aHR 2.58, 95%CI: 1.07–6.23).

**Conclusions:**

Our results showed that high NL ratio increased the risk of TB. NL ratio combined with TB symptoms could increase the accuracy of TB screening among HIV-infected individuals.

**Electronic supplementary material:**

The online version of this article (10.1186/s12879-019-4292-9) contains supplementary material, which is available to authorized users.

## Background

Tuberculosis (TB) is a major global diseases burden; in 2017, tuberculosis caused the highest number of unnecessary deaths from infectious diseases worldwide. Notably, individuals infected with human immunodeficiency virus (HIV) have a significantly higher risk of TB morbidity and mortality rates relative to HIV-uninfected individuals [1]. Thailand has a high burden of TB and HIV-TB co-infection. In 2016, HIV-infected individuals comprised 4764 (8%) of 68,040 total new and relapse TB cases, and 3900 HIV-associated TB deaths were reported in that year [1]. A previous study reported that half of all deaths occurring within 2 months of commencing TB treatment occurred among HIV-TB co-infected individuals due to difficulty in and delayed TB diagnosis [2]. Early detection of TB and preventive therapy is essential to reduce the morbidity and mortality from TB in HIV-infected individuals.

The World Health Organization (WHO) recommends TB screening for HIV-infected individuals to rule out active TB diseases before the initiation of preventive therapy [3, 4]. However, excluding active TB and early TB diagnosis using clinical symptoms and diagnostic tests remains challenging in HIV-infected individuals. The sensitivity of sputum microscopy ranges from 24 to 61% in HIV-infected individuals [5]. Further, uncommon clinical presentation [6, 7] and atypical chest X-ray presentation [8] in HIV patients makes TB diagnosis difficult. Although GeneXpert MTB/RIF assay was recommended as an initial diagnostic test in the WHO guideline, access to GeneXpert MTB/RIF is restricted and long-term sustainability without donor support is uncertain in resource-limited countries.

Recent studies have suggested that the neutrophil-to-lymphocyte ratio (NL ratio) has been associated with the severity or prognosis of several infectious diseases, such as community acquired pneumonia [9] and bacteremia [10, 11]. Moreover, a recent report from China indicated that high NL ratio in peripheral blood could predict the risk of pulmonary TB retreatment [12]. The NL ratio cannot be used as a specific marker to distinguish pulmonary TB from the other respiratory diseases. However, it could be an appropriate risk assessment tool and increase the accuracy of TB screening in resource-limited settings, as it is readily available from the routine hematological laboratory assessment at the point of entry to HIV care. We hypothesized that NL ratio was associated with the risk of TB infection among HIV-infected people. We aimed to investigate an association between NL ratio at TB screening and the incidence of active TB cases within 1 year after TB screening among HIV-infected adults in Thailand.

## Methods

### Study settings

In Thailand, day care centers (DCC) for HIV-infected individuals were established at the district hospitals in 1995 to support HIV treatment according to the national HIV/AIDS clinical guideline [13]. These centers provide social and psychological care [14]. Mae Chan hospital is a one of the large district hospitals in Chiang Rai province, which is located in the northernmost region of Thailand. In this hospital, the DCC works closely with the HIV and TB clinic to provide medical services. HIV-infected individuals are routinely introduced to the DCC at the hospital and are screened for TB based on clinical history, chest radiography, and sputum smear microscopic examination for acid fast bacilli (AFB) by trained laboratory technicians from the TB clinic. Culture and drug susceptibility testing is primarily performed in patients with positive sputum smear results after the samples are transferred to the microbiology laboratory at Chiang Rai hospital. In Chiang Rai province, isoniazid preventive therapy (IPT), as recommended in the national guideline, has been provided since 2001 to HIV-infected individuals who have positive tuberculin skin test (TST) results. However, only 9% of HIV-infected individuals received the IPT during this study period, because the initiation of IPT prophylaxis was dependent on the accessibility of isoniazid for preventive therapy in the hospitals and based on the decision of physicians.

### Study participants

Patients eligible for this prospective cohort study were HIV-infected individuals older than 18 years of age who had not previously received TB treatment and were registered at the DCC between 2002 and 2015. Patients who started IPT before the registration at the DCC and who started IPT after DCC registration were excluded from this analysis to avoid the intervention effect. Patients registered at the DCC received their TB screening at their first visit to the DCC. People who were diagnosed with active TB and were prescribed anti-TB treatment at the first TB screening were excluded from the analysis.

### Data collection

Baseline TB screening data were collected by trained research nurses at the DCC. A questionnaire was used to collect demographic information (age, sex, and ethnicity), recent TB symptoms, current and prior anti-retroviral treatment (ART), and TB treatment history (Additional file 1). Recent TB symptoms included: cough, night sweats, fever, weight loss, and hemoptysis within the last 4 weeks. All patients received 3-day sputum smear tests for AFB, chest X-ray, and blood laboratory tests at the hospital. When a sputum AFB smear result was positive, or if it was negative but from a patients with a high suspicion of TB, sputum samples were processed for *Mycobacterium tuberculosis* culture tests after being transferred to the microbiology laboratory in Chiang Rai hospital. The blood laboratory tests included CD4 cell counts, white blood cell counts, and differential counts. NL ratio was calculated from the absolute neutrophil counts divided by the absolute lymphocyte counts. Chest X-rays were interpreted for abnormal signs such as infiltrate, cavity, and pleural effusion by physicians who treated TB patients. TST was also performed during TB screening to identify latent TB infection. The skin test reaction was read 48–72 h after administration. A diameter of 5 or more millimeters in the indurated area was considered positive for latent TB infection [15]. The provincial-wide TB registry database was used to ascertain and validate the TB diagnosis and the treatment outcome, including mortality data. The provincial-wide TB registry database is a reporting system for TB, which records socio-demographic information, medication, treatment outcome, and laboratory examinations for all TB patients diagnosed in the TB clinic and the public hospital. TB cases were diagnosed based on clinical symptoms; and smear AFB results and TB culture results used for TB diagnosis could not be accessed during the follow-up.

### Definition of TB screening positive and development TB during follow-up

TB screening positive was defined by the initiation of TB treatment within 7 days after TB screening with AFB sputum positivity or positive *M. tuberculosis* culture at TB screening. Even if sputum AFB smear was negative within 7 days after TB screening, individuals diagnosed with TB by a physician and who started TB treatment based on clinical symptoms and/or chest X-ray results and/or culture positive results were defined as TB screening positive. The outcome of this analysis was the incidence of TB cases within 1 year of the first TB screening, after excluding TB screening positive cases. “New TB cases” was defined as TB patients who were registered in the provincial-wide TB registry system. The end of follow-up was the date of TB diagnosis, date of death, or 1 year after the first TB screening.

### Statistical analysis

Baseline characteristics at the first TB screening were summarized as median and interquartile range (IQR) for continuous variables and as frequency for categorical variables. Comparisons between positive TB screening and negative TB screening groups were tested using the chi-square test or Wilcoxon rank-sum test for categorical and continuous variables, respectively.

Univariate Cox-proportional hazards models were used to estimate the relationship between potential predictive variables and time-to-event outcome among individuals who screened negative for TB. The multivariate Cox proportional hazards model for the risk of TB diagnosis (including the variables (age, sex, ethnicity, year of DCC registration, ART status, TB symptoms, TST results, and chest X-ray results) assessed the effect of the NL ratio on the incidence of TB. Cubic-spline curves were used to assess possible nonlinear relationships between NL ratio and the outcome, and to determine the threshold of the NL ratio. The analysis was conducted using multivariate-adjusted cox-regression models and used 5 knots. The threshold level of NL ratio was identified on the cubic spline curve, which showed a statistically significant effect on TB development (adjusted hazard ratio > 1). The NL ratio threshold was used to categorize NL ratio into low or high. The effects of the categorized NL ratio (based on the threshold level) were analyzed in sub-groups (normal chest X-ray, TB symptoms and each CD4 count category). To determine the effect of NL ratio combined with TB symptoms and chest X-ray at the TB screening, we categorized participants into four groups (no-TB symptoms and normal chest X-ray, TB symptoms and normal chest X-ray, no-TB symptoms and abnormal chest X ray, and TB symptoms and abnormal chest X-ray) and assessed the effect of NL ratio on TB development. *P*-values were calculated using the likelihood ratio test. The threshold of statistical significance was a *P*-value of < 0.05. All analyses were conducted using STATA 14.0 (STATA Corp., College Station, Texas, USA).

## Results

### Baseline characteristics

A total of 1118 HIV-infected individuals (median age 35 years; 46.4% male) underwent TB screening with at least 3 sputum assays at the DCC between 2002 and 2015. Fifty-four patients diagnosed with active TB and who started TB medication were excluded from the subsequent analysis, based on the detection of at least 1 AFB positive smear (*N* = 8, 14.8%), TB culture positive (*N* = 40, 74.1%), or clinical symptoms highly suspected to be TB (*N* = 41, 75.9%). Patients that were TST positive, had abnormal chest X-rays, or reported TB symptoms were strongly associated with active TB compared to patients who were TB screening negative (Table 1). Baseline median NL ratio (median 3.27; IQR: 1.99–5.41) in active TB at the first TB screening were significantly higher than that in patients who were TB screening negative (median NL ratio 1.66; IQR: 1.14–2.54; *p* < 0.001).

**Table 1:** Baseline characteristics of HIV participants at the first TB screening

Variables	Category	Total cases of TB screening	TB screening^a^ positive for TB	TB screening negative for TB	*p*-value^†^
		*N* = 1118	*N* = 54	*N* = 1064	
Age (years)	Under 30	226 (20.2%)	9 (16.7%)	217 (20.4%)	0.71
	30–39	506 (45.3%)	24 (44.4%)	482 (45.3%)	
	Over 40	386 (34.5%)	21 (38.9%)	365 (34.3%)	
Sex	Male	510 (45.6%)	37 (68.5%)	473 (44.4%)	0.002
	Female	604 (54.0%)	17 (31.5%)	587 (55.2%)	
	Missing data	4 (0.4%)	–	4 (0.4%)	
Ethnicity	Thai	782 (69.9%)	34 (63.0%)	748 (70.3%)	0.50
	Hill tribe	211 (18.9%)	13 (24.0%)	198 (18.6%)	
	Others	125 (11.2%)	7 (13.0%)	118 (11.1%)	
Year of DCC registration	2002–2006	489 (43.7%)	22 (40.7%)	467 (43.9%)	0.48
	2007–2011	500 (44.7%)	23 (42.6%)	477 (44.8%)	
	2012–2015	129 (11.6%)	9 (16.7%)	120 (11.3%)	
CD4 (/mm^3^)	< 50	262 (23.4%)	20 (37.0%)	242 (22.7%)	< 0.001
	50–199	292 (26.1%)	23 (42.6%)	269 (25.3%)	
	≧200	564 (50.5%)	11 (20.4%)	553 (52.0%)	
ART	No	633 (56.6%)	41 (75.9%)	592 (55.6%)	0.008
	Past/Current	335 (30.0%)	5 (9.3%)	330 (31.0%)	
	Start during follow-up	130 (11.6%)	7 (13.0%)	123 (11.6%)	
	Missing data	20 (1.8%)	1 (1.8%)	19 (1.8%)	
TST (Positive: > 5 mm)	Negative	981 (87.7%)	36 (66.7%)	945 (88.8%)	< 0.001
	Positive	94 (8.4%)	15 (27.8%)	79 (7.4%)	
	Not tested	43 (3.9%)	3 (5.6%)	40 (3.8%)	
Abnormal chest X ray	No	831 (74.3%)	23 (42.6%)	808 (75.9%)	< 0.001
	Yes	281 (25.1%)	31 (57.4%)	250 (23.5%)	
	No tested	6 (0.5%)	–	6 (0.6%)	
TB symptoms^b^	No	693 (62.0%)	13 (24.1%)	680 (63.9%)	< 0.001
	At least one symptom	425 (38.0%)	41 (75.9%)	384 (36.1%)	
Whole blood cell counts (/mm^3^)	Median (IQR)	5700 (4400–7100)	5970 (4800–7500)	5700 (4400–7100)	0.30
Lymphocyte counts (/mm^3^)	Median (IQR)	1733.7 (1176.7–2324.7)	1170.5 (752.1–1596.7)	1777.0 (1196.2–2346.8)	< 0.001
Monocyte counts (/mm^3^)	Median (IQR)	343.1 (234.5–485.9)	374.0 (220.0–500.9)	341.5 (235.2–483.7)	0.53
Neutrophil counts (/mm^3^)	Median (IQR)	2877.9 (2160.0–3939.0)	3585.9 (2880.0–5079.6)	2827.3 (2143.6–3884.8)	< 0.001
NL ratio	Median (IQR)	1.67 (1.16–2.64)	3.27 (1.99–5.41)	1.66 (1.14–2.54)	< 0.001

*TB* Tuberculosis, *ART* Anti-retroviral treatment, *TST* Tuberculin skin test, *IQR* Interquartile range, *NL* Neutrophil to lymphocyte.

^a^TB screening was conducted by sputum acid-fast bacilli (AFB) positive, clinical symptoms, chest X-ray, TST, and sputum culture test

^b^TB symptoms include cough, night sweats, fever, weight loss, and hemoptysis within 4 weeks

^†^*p*-values were calculated using the chi-square test for categorical variables and Wilcoxon rank-sum test for continuous variables

### New TB cases after TB screening

**Fig. 1:**
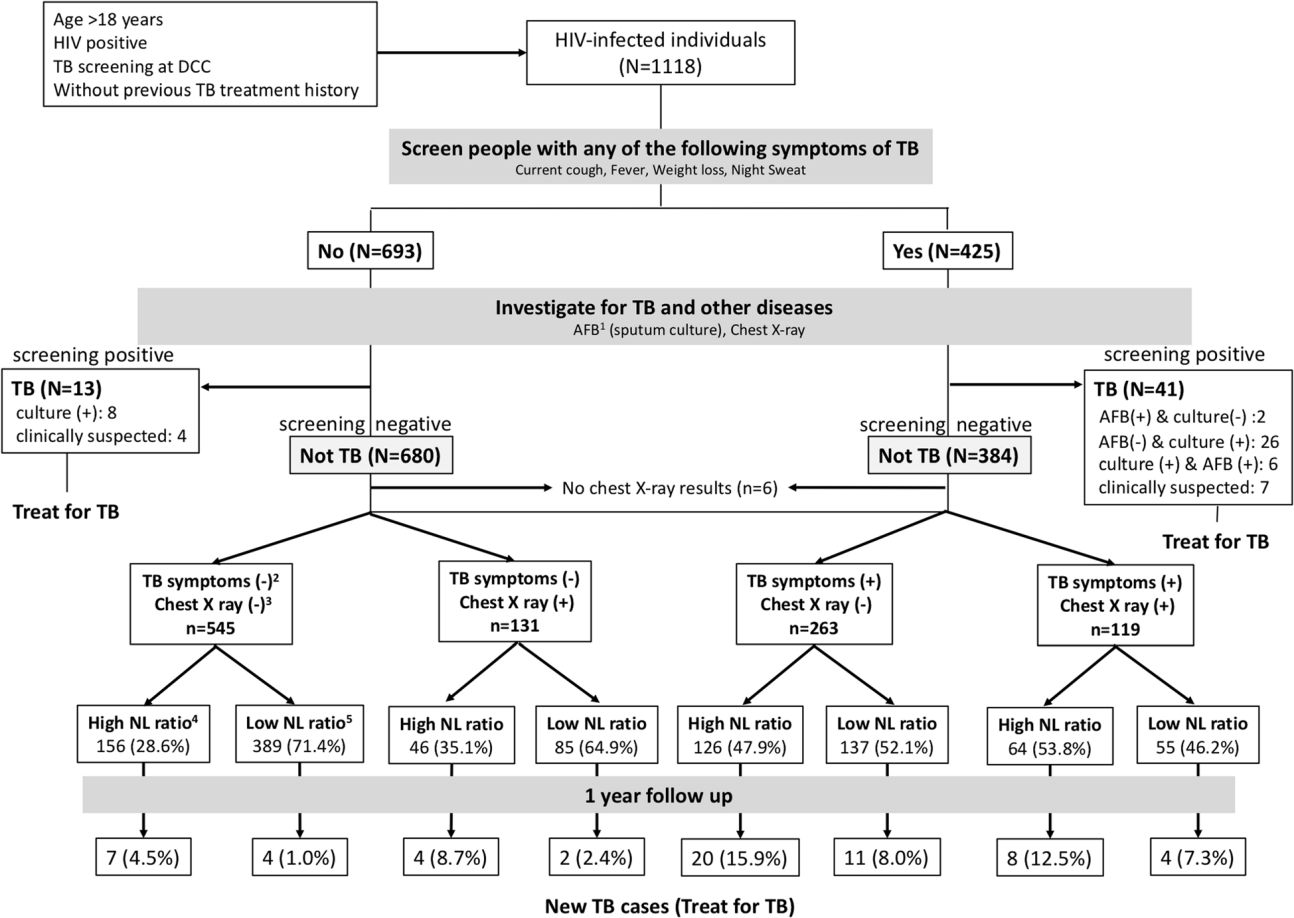
Flowchart of study participants and clarification of subgroups in day care center of Mae Chan hospital, Chiang Rai, Thailand. 1. AFB: 3-day sputum acid-fast bacilli results, 2. TB symptoms include cough, night sweats, fever, weight loss, and hemoptysis within 4 weeks. “TB symptoms (-)” refers to no TB symptoms and “TB symptoms (+)” refers to individuals with at least one TB symptoms. 3. “Chest X ray (-)” means normal chest X-ray. “Chest X-ray (+)” means that the chest X-ray showed some abnormal findings. 4. High NL ratio (neutrophil-to-lymphocyte ratio > 2), 5. Low NL ratio (neutrophil-to-lymphocyte ratio ≦2)

**Fig. 2:**
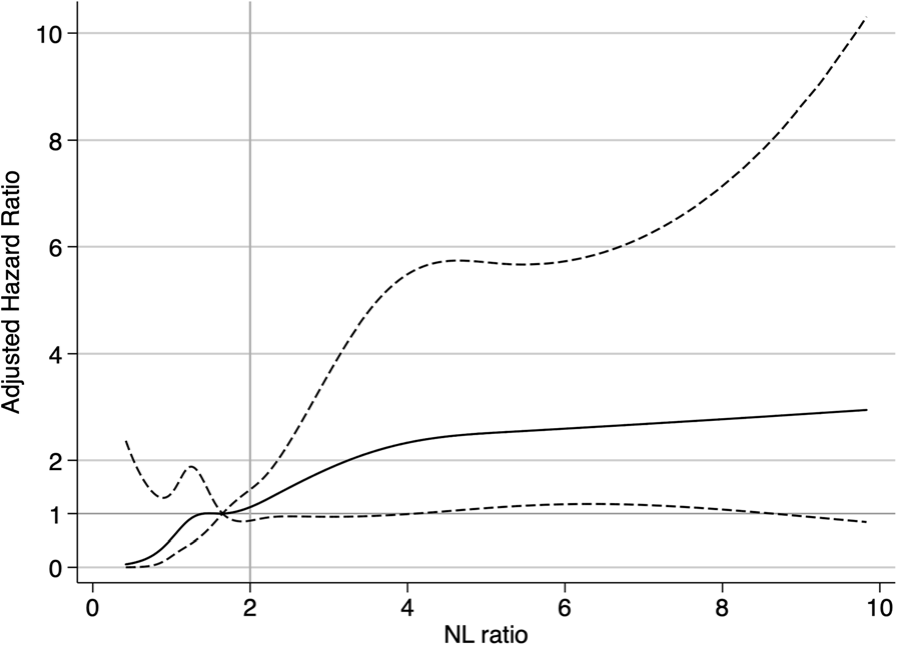
Multiple-adjusted hazard ratio and 95% confidence intervals for TB development associated with neutrophil-to-lymphocyte ratio (NL ratio). Hazard ratio of **TB development** with **NL ratio** adjusted for age, sex, ethnicity, year of registration, CD4 counts, ART, TST results, abnormal chest x-ray, and TB symptoms. The straight line shows the adjusted hazard ratio and the dashed line shows the 95% confidence intervals from the restricted cubic spline model. NL ratio was trancated at the 1st and 99th percentile (0.41 and 10, respectively). The median of NL ratio (1.65) was used as a reference. The gray straight line shows the threshold value (NL ratio = 2)

Of 1064 individuals who were TB screening negative (916.23 person-years follow-up), 5.6% (*n* = 60) of patients developed TB (incidence rate: 65.5 per 1000 person-year, 95% CI: 50.9–84.3) and 78.3% (*n* = 47) of them were diagnosed within 6 months after the first TB screening (Fig. 1). Among the 60 patients who were diagnosed with TB after the first TB screening, 26 died within 6 months of TB diagnosis (median 31 days, IQR: 10–68 days). After adjusting for age, sex, ethnicity, year of registration, CD4 counts, TB symptoms, abnormal chest x-ray, TST results and NL ratio, low CD4 counts (< 200/mm^3^), positive TST, TB symptoms and male sex were found to increase the risk of TB development. TST positive patients had a 3.5-fold higher risk of becoming a new TB case. However, the proportion of TST positive patients attributable to TB cases was only 10.1%. A total of 330 (31.0%) patients commenced ART before TB diagnosis, while 123 (11.6%) patients started ART after or at the time of TB diagnosis. After adjusting for age, sex, ethnicity, year of DCC registration, CD4 counts, ART, TST results, chest X-ray and TB symptoms (Table 2), each unit difference of NL ratio corresponded to a 1.06-fold higher risk of TB development. Multivariable-adjusted restricted cubic spline analysis (Fig. 2) suggested that the aHR of TB diagnosis gradually increased corresponding to increasing NL ratio. NL ratio above 2 was the threshold level for a significant effect on TB development.

**Table 2:** Risk factors of development TB within 1 year of TB screening among 1064 HIV infected, TB screening negative adults at the Mae Chang day care center

Variables	Category	TB screening negative for TB	New TB cases	Hazard Ratio (95% CI)	Adjusted Hazard Ratio (95% CI)	*p*-value^†^
		*N* = 1064	*N* = 60			
NL ratio	per unit	1.66 (1.14–2.54)	2.63 (1.68–4.53)	1.13 (1.08–1.18)	1.06 (1.00–1.13)	0.069
Age (years)	Under 30	217	14 (6.5%)	1.02 (0.54–1.93)	1.30 (0.68–2.48)	0.391
	30–39	482	30 (6.2%)	reference	reference	
	Over 40	365	16 (4.4%)	0.68 (0.37–1.25)	0.77 (0.41–1.45)	
Sex	Male	473	37 (7.8%)	2.06 (1.22–3.46)	1.76 (1.02–3.05)	0.039
	Female	587	23 (3.9%)	reference	reference	
	Missing data	4	0	–		
Ethnicity	Thai	748	40 (5.4%)	reference	reference	0.281
	Hill tribe	198	15 (7.6%)	1.36 (0.75–2.46)	1.29 (0.69–2.42)	
	Others	118	5 (4.2%)	0.80 (0.32–2.04)	0.59 (0.22–1.54)	
Year of DCC registration	2002–2006	467	28 (6.0%)	reference	reference	0.259
	2007–2011	477	27 (5.7%)	0.87 (0.51–1.47)	0.78 (0.44–1.39)	
	2012–2015	120	5 (4.2%)	0.64 (0.25–1.65)	0.45 (0.16–1.25)	
CD4 counts (cells/mm^3^)	< 50	242	25 (10.3%)	7.53 (3.61–15.71)	4.20 (1.75–10.08)	< 0.001
	50–199	269	25 (9.3%)	5.78 (2.77–12.03)	4.36 (2.00–9.50)	
	≧200	553	10 (1.8%)	reference	reference	
ART	No	592	40 (6.8%)	reference	reference	0.357
	Past/Current	330	12 (3.6%)	0.49 (0.25–0.92)	0.71 (0.35–1.44)	
	Start during follow-up	123	7 (5.7%)	0.83 (0.38–1.86)	0.51 (0.21–1.20)	
	Missing data	19	1 (5.3%)	0.69 (0.09–5.01)	1.53 (0.20–11.78)	
TST (Positive: > 5 mm)	Negative	945	51(5.4%)	reference	reference	0.019
	Positive	79	8 (10.1%)	1.88 (0.89–3.96)	3.52 (1.58–7.84)	
	Not tested	40	1 (2.5%)	0.54 (0.07–3.88)	0.55 (0.08–4.05)	
Abnormal X ray	No	808	42 (5.2%)	reference	reference	0.800
	Yes	250	18 (7.2%)	1.47 (0.85–2.56)	1.08 (0.61–1.90)	
	No tested	6	0	–	–	
TB symptoms^b^	No	680	17 (2.5%)	reference	reference	< 0.001
	At least one symptom	384	43 (11.2%)	5.19 (2.96–9.11)	3.14 (1.70–5.79)	

*TB* Tuberculosis, *CI* Confidence interval, *NL* Neutrophil to lymphocyte, *DCC* Day care center, *ART* Anti-retroviral treatment, *TST* Tuberculin skin test

^a^TB symptoms include cough, night sweats, fever, weight loss, and hemoptysis within 4 weeks

^†^*p*-values were calculated using likelihood ratio test

### NL ratio

Categorized NL ratios (high: NL ratio > 2 and low: NL ratio ≦2) were analyzed to examine whether the NL ratio was associated with outcomes in different conditions (TB symptoms, chest X-ray and CD4 count categories) (Fig. 1). A high NL ratio corresponded to a 2.2-folds higher risk of progression to active TB compared to low NL ratio among all HIV-infected individuals with negative TB screening. Even among the normal chest X-ray group, high NL ratio was a high risk for active TB. Moreover, among the normal chest X-ray group, combined TB symptoms with a high NL ratio could predict a 2.5-times higher risk of TB development compared to a low NL ratio combined with TB symptoms. When stratified by CD4 counts, the association between high NL ratio and TB disease was stronger among the lowest CD4 count group (CD4 counts < 50/mm^3^) compared to the group with higher CD4 counts (≧50/mm^3^), (Table 3).

**Table 3:** TB disease development associated with the ratio of neutrophil to lymphocyte (NL ratio) in sub-groups

		Total	Cases	Univariate analysis		Multivariate analysis^a^	
				Hazard Ratio (95% CI)	*p*-value	Adjusted Hazard Ratio (95% CI)	*p*-value
All (*n* = 1064)	Low NL ratio	670	21 (3.1%)	Reference	< 0.001	Reference	0.007
	High NL ratio	394	39 (9.9%)	3.75 (2.21–6.38)		2.19 (1.23–3.90)	
TB symptoms (−) + Chest X ray (−) (*n* = 545)	Low NL ratio	389	4 (1.0%)	Reference	0.014	Reference	0.276
	High NL ratio	156	7 (4.5%)	4.68 (1.37–16.00)		2.10 (0.55–7.97)	
TB symptoms (−) + Chest X ray (+) (*n* = 131)	Low NL ratio	85	2 (2.4%)	reference	0.074	reference	0.264
	High NL ratio	46	4 (8.7%)	4.71 (0.85–25.82)		3.08 (0.43–22.26)	
TB symptoms (+) + Chest X ray (−) (*n* = 263)	Low NL ratio	137	11 (8.0%)	Reference	0.016	Reference	0.035
	High NL ratio	126	20 (15.9%)	2.47 (1.18–5.18)		2.58 (1.07–6.23)	
TB symptoms (+) + Chest X ray (+) (*n* = 119)	Low NL ratio	55	4 (7.3%)	Reference	0.201	Reference	0.359
	High NL ratio	64	8 (12.5%)	2.19 (0.66–7.31)		1.90 (0.48–7.53)	
CD4 counts: < 50 (cells/mm^3^) (*n* = 242)	Low NL ratio	71	4 (5.6%)	Reference	0.062	Reference	0.045
	High NL ratio	171	21 (12.3%)	2.76 (0.94–8.07)		3.17 (1.03–9.80)	
CD4 counts: 50–199 (cells/mm^3^) (*n* = 269)	Low NL ratio	155	10 (6.3%)	Reference	0.043	Reference	0.084
	High NL ratio	114	15 (8.8%)	2.28 (1.03–5.10)		2.10 (0.90–4.90)	
CD4 counts: > 200 (cells/mm^3^) (*n* = 553)	Low NL ratio	444	7 (1.6%)	Reference	0.402	Reference	0.301
	High NL ratio	109	3 (2.8%)	1.78 (0.46–6.89)		2.22 (0.49–10.15)	

*TB* Tuberculosis, *HR* Hazards ratio, *CI* Confidence interval, *NL* Neutrophil to lymphocyte

^a^ HR adjusted for age, sex, ethnicity, year of registration, CD4 counts, ART, TST results, abnormal chest x-ray, and TB symptoms

## Discussion

In this Thai cohort study, 5.6% of HIV-infected individuals without TB at the first TB screening were diagnosed with TB within 1 year. Approximately half of these patients died after TB diagnosis and treatment. A high NL ratio was observed in HIV-infected individuals who were TB screening positive at the first TB screening. As NL ratio is an inflammatory marker associated with other infectious diseases, cancers and cardiovascular diseases [10, 16, 17], this ratio is not a TB-specific biomarker. Nevertheless, our results showed that NL ratio combined with TB symptoms could predict the risk of new TB cases even if HIV-infected individuals had a normal chest X-ray findings and AFB smear negative results.

The association between NL ratio and TB has been suggested in previous studies. Yin et al. reported that high NL ratio (cutoff value ≧2.53) at pretreatment was associated with a 2.41- times higher odds of TB retreatment (adjusted odds ratio = 2.409, 95% CI: 1.212–4.788) [12]. A study from sub-Saharan Africa by Sutherland et al. demonstrated that the granulocyte/lymphocyte ratio improved the sensitivity of diagnosis from 67 to 93% with the correct classification into TB and latent TB, based on a comparison of using the proportions of granulocytes alone [18]. Our findings supported these results and provided new perspectives, especially in HIV patients. Another study, conducted in Korea, suggested that NL ratio had high sensitivity and specificity for differentiating between pulmonary TB and bacterial community-acquired pneumonia [19]. High NL ratio (> 7) was suggested to be the optimal cut-off point to discriminate TB patients from cases of community acquired pneumonia. This study suggested that the threshold level of NL ratio > 2 cannot be used a specific marker to differentiate TB from the other respiratory infectious diseases. Additional research may be needed to assess the optimal cut-off point for differentiating between pulmonary TB and other respiratory infectious diseases at TB screening among HIV-infected individuals.

A high NL ratio represents elevated neutrophils and/or decreased lymphocytes. In the early phase of *M. tuberculosis* infection, neutrophils are the predominant infected white blood cell, with involvement of granuloma formation [20] or pulmonary destruction [21]. High neutrophil counts are correlated with high mortality [22], sputum *M. tuberculosis* positivity [23], and delay in smear negative conversion [24]. Furthermore, lymphocytes, especially T-lymphocyte subsets, are important immune cells against TB infections. Lymphocyte counts are negatively correlated with clinical TB severity [21, 25]. Several studies have demonstrated that the percentage of peripheral blood T-cell subsets (CD4+ and CD8+) in active TB patients were significantly decreased compared to healthy controls, with reduced numbers of total and central memory CD4 + T cells [26]. In addition to the specific roles of neutrophils and lymphocytes, the interaction between these cells plays an important role in the innate and adaptive immunity response to *M. tuberculosis*. Several studies have suggested that neutrophils suppress mycobacterial-specific T cell activation [27, 28]. Further studies are required to clarify how the NL ratio relates to the mechanism of TB development; for example, whether NL ratio increases as a result of TB infection or whether it can be used to identify HIV-infected individuals who are at a high risk for progressing to active TB. Monocyte counts and monocyte-to-lymphocyte ratio (ML ratio) were also reported as a marker of TB development in previous studies [29–31]. Our study showed that monocyte counts have no association with TB cases at the initial screening. The counts and ML ratio may vary depend on the TB stages, severity of diseases, and background of the population.

HIV-infected individuals are at increased risk of death, especially in countries with high prevalence of TB and HIV, due to delay in initiation of TB treatment, immunosuppression, and lack of laboratory confirmation for accurate TB diagnosis, which may result in misdiagnosis and inaccurate treatment for TB [5, 32–34]. Current standard diagnosis using AFB staining and clinical manifestations, cannot always accurately detect active TB cases, especially in HIV-infected individuals with unordinary clinical manifestations and low AFB positivity. In our study, the high number of new cases within 1 year of TB screening suggested that a high number of patients were being misdiagnosed at the first TB screening. Rapid point of care diagnostic tools, such as the gene Xpert MTB/RIF assay or the urine TB-LAM urine antigen test, are reported to be useful for higher yield and sensitivity, compared to sputum smear microscopy at TB screening [35–38]. However, the high cost of these methods may be prohibitive for use in daily clinical practice in resource-limited settings. After adjusting for the predictive factors of active TB (age, sex, ethnicity, TB symptoms, low CD4 counts, abnormal chest X-ray, and TST positive result), high NL ratio increased the risk of developing active TB in our study. If these individuals were targeted for early therapy, enhanced follow-up, or additional high-sensitivity diagnostic tests such as gene Xpert MTB/RIF assay, this could potentially reduce the development of TB and associated mortality.

This study has several limitations. Firstly, TB screening and TB diagnosis were not performed using sensitive methods, such as a sputum culture test or the gene Xpert MTB/RIF assay, reflecting the practical limitations in Thailand during this study period (2002–2015). Therefore, there may have been cases of missed diagnosis or over diagnosis both at TB screening and during the follow-up period. Moreover, non-tuberculosis mycobacteria infections or pneumonia might be misclassified as TB cases in this cohort of HIV-infected patients. Secondly, we could not include the timing of HIV diagnosis and the date of ART initiation, due to lack of such data. The timing of HIV testing and ART initiation might be associated with the timing of TB screening and incidence of active TB. Thirdly, we could not determine when patients became infected with TB (after or before the initial TB screening). Based on the high number of patients who developed TB within 6 months of the initial TB screening, we suspect that most of the new TB cases were infected with TB but could not be diagnosed with TB at the initial screening, as these cases were all sputum smear negative and diagnosed without high sensitivity diagnostic tools. We could not estimate the proportion of cases that were misdiagnosed at the initial TB screening. Finally, we did not consider other opportunistic infections that might have affected the neutrophil and lymphocyte counts. There is high possibility that some patients were co-infected with other infectious diseases. Therefore, an additional study excluding the effect of other infectious diseases is necessary to confirm this finding.

## Conclusions

NL ratio was associated with active TB at initial TB screening, and predicted the risk of TB among HIV-infected individuals. NL ratio could improve the accuracy of TB screening and assist in earlier TB diagnosis. Furthermore, NL ratio could be used to identify patients who required further follow-up, even if they were negative at TB screening. Therefore, NL ratio could be a useful and cost-effective indicator for assessing the risk of TB among HIV-infected individuals especially in resource-limited settings.

## Additional file

Additional file 1: Questionnaire. (PDF 355 kb)

## Data Availability

The datasets used and/or analyzed during the current study are available from the corresponding author on reasonable request.

## Abbreviations

(a)HR: (adjusted) Hazard ratio
AFB: Acid fast bacilli
ART: Antiretroviral therapy
DCC: Day care center
HIV: Human immunodeficiency virus
IPT: Isoniazid preventive therapy
IQR: Interquartile range
NL ratio: Neutrophil to lymphocyte ratio
TB: Tuberculosis
TST: Tuberculin skin test
WHO: World Health Organization
