# Supplementary material for: Predicting the risk of pulmonary tuberculosis based on the neutrophil-to-lymphocyte ratio at TB screening in HIV-infected individuals
Source: BMC Infect Dis. 2019 Jul 29;19:667. doi: 10.1186/s12879-019-4292-9 (PMC6664723; doi:10.1186/s12879-019-4292-9)
Supplement: Supplementary file 1 — Questionnaire. (PDF 355 kb) [file 12879_2019_4292_MOESM1_ESM.pdf]

QUESTIONNIARE

Project Title: Evaluation of impact of Tuberculosis prevention on TB morbidity and mortality for people living with HIV/AIDS (PLWHA) and cross-sectional assessment care for PLWHA registered at adult day care center in Chiang Rai province

INSTRUCTION Please fill up every items and check whether the data are complete before doing data entry

Interviewer…………………………………………… Interview date……………………………….

Interview place……………………………………… At what hospital……………………………..

QN inspector…………………………………. Date…………………………………………..

Data entry 1………………………………… Date…………………………………………..

Data entry 2………………………………… Date…………………………………………..

Gender [ ] Male [ ] Female Age__________years

- HIV testing with positive result firstly at the hospital in the area under control (from OPD card, counseling room, and so on that has approved document)

  [ ] Unavailable [ ] Available on Date………/…………/……..…… code……………….……...

- HIV testing at other hospitals (from OPD card, laboratory result report, medical certificate)

  [ ] Unavailable [ ] Available on Date………/…………/……..…… code……………….……...

  HIV testing place ………………………………………………

Part 1. General health and risk factors

Weight……………………….…………………kg. Height………………cm.

Blood pressure………………..………mm.Hg. Pulse………………….times/min,

Body temperature……………………………..^{o}C (if having fever)

1. Physical examination

1.1 BCG scar [ ] 1. No [ ] 2.Yes [ ] 3. Not sure

1.2 General appearance [ ] 1. Normal [ ] 2. Abnormal (specify)……………………….………

1.3 EENT (mouth/tongue) [ ] 1. Normal [ ] 2. Abnormal (specify)…………………….…………

1.4 Chest/Lung [ ] 1. Normal [ ] 2. Abnormal (specify)……………………

1.5 Abdomen [ ] 1. Normal [ ] 2. Abnormal (specify)……………………

1.6 Neurological [ ] 1. Normal [ ] 2. Abnormal (specify)……………………

1.7 Skin [ ] 1. Normal [ ] 2. Abnormal (specify)……………………

1.8 Others (specifiy)………………………………………………………………………

2. What symptoms bring you to the hospital?

2.1 Cough [1] No [2]Yes, how long? ………..day(s)

Explain the pattern of cough, frequency, and physical characteristics of the sputum………………

2.2 Hemoptysis [1] No [2] Yes 2.3 Chest pain [1] No [2] Yes

2.4 Fatigue [1] No [2] Yes 2.5 Anorexia [1] No [2] Yes

2.6 Fever [1] No [2] Yes

2.7 Weight loss [1] No [2] Yes, decreased for……….kg in……..…..month(s)

2.8 Others [1] No [2] Yes, specify……………………………………….

3. With what ability can you complete your daily activities?

[1] Excellent, able to do activities and work as usual

[2] Fair, able to work but less than usual, able to do activities as usual

[3] Poor, could not work, need care taker for daily activities

[4] Could not do anything, need care taker

Karnofsky score……………………

4. Have you get these symptoms during the past 6 months?

4.1 Cough more than 2 weeks	[1] No	[2]Yes, how long ………..day(s) when….
4.2 Hemoptysis	[1] No	[2] Yes
4.3 Chest pain	[1] No	[2] Yes
4.4 Night sweating	[1] No	[2] Yes
4.5 Fatigue	[1] No	[2] Yes
4.6 Anorexia	[1] No	[2] Yes
4.7 Weight loss	[1] No	[2] Yes, decreased for… kg in ………months
4.8 Persistent fever	[1] No	[2] Yes
4.9 Lymphadenopathy	[1] No	[2] Yes
4.10 Others	[1] No	[2] Yes, specify………………………………

*** Had you got these symptoms during the past 1 month?***

(1) Cough more than 2 weeks	[1] No	[2]Yes, how long ………..days, when………………
(2) Hemoptysis	[1] No	[2] Yes
(3) Chest pain	[1] No	[2] Yes
(4) Night sweating	[1] No	[2] Yes
(5) Fatigue	[1] No	[2] Yes
(6) Anorexia	[1] No	[2] Yes
(7) Weight loss	[1] No	[2] Yes, decreased for…….. kg in………months
(8) Persistent fever (need to take medicine or tepid sponge)	[1] No	[2] Yes
(9) Lymphadenopathy	[1] No	[2] Yes
(10) Other symptoms ………...	[1] No	[2] Yes specify……………………………………

5. Have you ever had these opportunistic infections?

5.1 Pneumocystis carinii Pneumonia (PCP)	[1] No	[2] Yes when………………
5.2 Cryptococcus meningitis	[1] No	[2] Yes when………………
5.3 Oral candidiasis	[1] No	[2] Yes when………………
5.4 Chronic diarrhea (> 1 month)	[1] No	[2] Yes when………………
5.5 Herpes zoster	[1] No	[2] Yes when………………
5.6 Herpes simplex	[1] No	[2] Yes when………………
5.7 Pruritic Papular Eruption (PPE)	[1] No	[2] Yes when………………
5.8 Persistent fever (> 2 weeks)	[1] No	[2] Yes when………………
5.9 Toxoplasmosis	[1] No	[2] Yes when………………
5.10 Others (specify)…………………………..	[1] No	[2] Yes when………………

Since being infected with HIV, How was the symptoms of the most serious illness you ever had? Did you have to admit in the hospital and when was it?

……………………………………………………………………………………………………

……………………………………………………………………………………………………

……………………………………………………………………………………………………

……………………………………………………………………………………………………

……………………………………………………………………………………………………

The current symptoms recorded for seeing the doctor (CC.)

……………………………………………………………………………………………………

……………………………………………………………………………………………………

……………………………………………………………………………………………………

……………………………………………………………………………………………………

……………………………………………………………………………………………………

6. Do you drink?

[1] never

[2] used to drink but had already stopped drinking (at least for 6 months), when……………..

[3] still drinking within past 6 months ( ) at least 4 days / week ( ) less than 4 days / week

7. Do you smoke?

[1] Never

[2] Used to smoke but had already stopped smoking (at least for 6 months), when……………….

[3] Still smoking for ……………cigarette(s)/day

8. In the past, did you use any illegal drugs?

[1] Never

[2] Used to use but had already stopped (at least for 6 months), when…………………

Specify the type of drug ………………..……by ☐ smoking ☐ injection ☐ others, specify…….

[3] Still using the drugs,

Specify the type of drug ……………………..…by ☐ smoking ☐ injection ☐ others, specify…...

9. Have you ever been a prisoner?

[1] Never [2] yes, when..………………… and how long ....year(s).......month(s)……...day(s)

10. Are you having or being treated diabetes mellitus?

[1] No [2] Yes, have been treating for……………years [3] Do not know/ Not sure

11. Do you have underlying disease(s) under treatment by medicine, for example, hypertension, asthma, and emphysema?

[1] No [2] Yes, specify the disease/symptom…………being treated at…….……..

12. When had you ever tested and known the HIV result? Date……/……../………….. Tested at………..

13. Risk factor for HIV infection

(1) Sexual transmission (2) Drug injection (3) Blood transfusion (4) Mother to child transmission

14. After knowing your first HIV test result for ………………..month(s) you decided to be a member of Day care center at the hospital

14.1 Are you a member of a sub-district Day care group? [ ] No [ ] Yes, group name…………………..…

15. Has a doctor ever told you that you have tuberculosis?

[1] No

[2] Yes, from what hospital…………………….…….., when……………… , treated for………month

Summary 2.1) still on TB treatment

2.2) used to have TB but completed the treatment course

Data from medical records (OPD card)

TB No. ………..Date of TB registry……………Date of complete treatment course…………..……

TB type…………..sputum result before start treatment………..…treatment outcome……………

( ) no data in medical record (OPD card)

[3] Not sure

16. Do you have any relatives currently or used to have TB?

[1] No, skip to No. 17 [2] Yes, in relationship with you as …………………………

The hospital he/she treated TB…………….. when (date)……………. for ……months

Current status of TB treatment …… (2.1) still on TB treatment

…… (2.2) used to have TB but completed the treatment course

Data from medical records (OPD card)

TB No. ………..Date of TB registry……………Date of complete treatment course…………..……

TB type…………..sputum result before start treatment………..…treatment outcome……………

( ) no data in medical record (OPD card)

[3] Not sure

16.1 In case you answer YES in No. 16, do you live in the same house of the TB patient?

[ ] 1. No, skip to no. 16.4 [ ] 2. Yes

16.2 In case you answer YES in No. 16.1, do you share your bed room with the TB patient?

[1] No [2] Yes, for………………….. days (from having TB symptoms to TB treatment completion)

16.3 In case you answer YES in No. 16.2, do you share your bed with the TB patient?

[1] No [2] Yes

16.4 In case you answer NO in No.16.1, had you ever taken care of the TB patients during their illness?

[1] No [2] Yes, taking care activities.……………………………..…………………………

Frequency………………………….. hr/day (average) or ……………. Day(s)/week

Since when did you take care the patients (specify clearly if it was before or after the treatment with anti-TB medicine)…………………………………………………

17. Have you ever taken care of your friend or your acquaintance including your husband or wife during their illness from TB?

[1] No [2] Yes, in relationship with you as …………………………

The hospital he/she treated TB…………….. when (date)……………. for ……months

Current status of TB treatment …… (2.1) still on TB treatment

…… (2.2) used to have TB but completed the treatment course

Data from medical records (OPD card)

TB No. ………..Date of TB registry……………Date of complete treatment course…………..……

TB type…………..sputum result before start treatment………..…treatment outcome……………

( ) no data in medical record (OPD card)

[3] Not sure

17.1 In case you answer YES in No.17, do you stay in the same house of the TB patient?

[1] No, skip to No. 17.4 [2] Yes

17.2 In case you answer YES in No. 17.1, do you share your bed room with the TB patient?

[1] No

[2] Yes, for………………….. days (from having TB symptoms to complete TB treatment)

17.3 In case you answer YES in No. 17.2, Do you share your bed with the TB patient?

[1] No

[2] Yes

17.4 In case you answer NO in No.17.1, had you ever taken care of the TB patients during their illness?

[1] No

[2] Yes, taking care activities.………………………………………………………………

Frequency………………………….. hr/day (average) or ……………. Day(s)/week

Since when did you take care the patients (specify clearly if it was before or after the the treatment with anti-TB medicine…………………………………………………...………………………………..…

General remark, interesting data from the interview

……………………………………………………………………………………………………

……………………………………………………………………………………………………

……………………………………………………………………………………………………

……………………………………………………………………………………………………

Part 2. Preventive therapy for opportunistic infections and ARV (data from the interview)

1. Isoniazid preventive therapy (IPT)

[ ] 1. No

[ ] 2. Used to have, but stopped. Start date…………………..from hospital…………………………..…

Date stopped………………………..reason for stopping…………………………………………..

[ ] 3. Yes, start date………from hospital…… now you are being prescribed isoniazid at hospital…….

In case you have IPT, how do you take it?

[1] Continuously take the isoniazid

[2] Forget to take the isoniazid sometimes, how …………………………………………………

Remark……………………………………………………………………………………….

2. PCP prevention (Cotrimoxazole/ Dapsone)

[ ] 1. No

[ ] 2. Used to have, but stopped. Start date…………………..from hospital…………………………..…

Date stopped………………………..reason for stopping…………………………………………..

[ ] 3. Yes, start date………from hospital…… now you are being prescribed isoniazid at hospital…….

In case you have cotrimoxazole/dapsone, how did you take it?

[1] Continuously take it

[2] Forget to take it sometimes, how …………………………………………………

Remark……………………………………………………………………………………….

3.Cryptococcus meningitis therapy (Fluconazole)

[ ] 1. No

[ ] 2. Used to have, but stopped. Start date…………………..from hospital…………………….…

Date stopped……………………..reason for stopping……………………………………..

[ ] 3. Yes, start date………from hospital…… now you are being prescribed isoniazid at hospital……..

In case you have cotrimoxazole/dapsone, how did you take it?

[1] Continuously take it

[2] Forget to take it sometimes, how …………………………………………………

Remark……………………………………………………………………………………….

4. Antiretroviral drug (ARV)

[ ] 1. No, skip to part 3

[ ] 2. Yes,

☐ 2.1 Used to be prescribed ARV but had already stopped the ARV, not taking any ARV now

☐ 2.2 On ARV and previously have the records of ARV prescribed or ARV stop

☐ 2.3 On ARV and does not have the record of ARV prescribed or ARV stop (NAÏVE)

ARV treatment records

• Date for 1st time of ARV prescription……………………...……...………………

Start from [ ] hospital…………… [ ] PHPT………………... [ ] others, specify………………

CD4 result before being prescribed ARV…………..%..............cells, Lab date …/……/………

ARV regimen [1] GPO virs 30, 40 (3TC+d4T+NVP) [2] GPO virZ 250 (3TC+AZT+NVP)

[3] 3TC (Lamivudine) [4] Combid (3TC+AZT)

[5] AZT (Zidovudine) [6] IDV (Indinavir / Crixivan)

[7] d4T (Stavudine) [8] RTV (Ritronavir / Norvir)

[9] ddI [10] SQV (Saquinavir)

[11] NVP (Nevirapine) [12] EFV (Efavirenz)

[13] others……………………….

Remark...

…………………………………………………………………………………………

Date of stopping or changing regimen………..…reason…………………………………………

• Date for 2nd time of ARV prescription……………………...……...………………

Start from [ ] hospital…………… [ ] PHPT………………... [ ] others, specify………………

CD4 result before being prescribed ARV…………..%..............cells, Lab date …/……/………

ARV regimen [1] GPO virs 30, 40 (3TC+d4T+NVP) [2] GPO virZ 250 (3TC+AZT+NVP)

[3] 3TC (Lamivudine) [4] Combid (3TC+AZT)

[5] AZT (Zidovudine) [6] IDV (Indinavir / Crixivan)

[7] d4T (Stavudine) [8] RTV (Ritronavir / Norvir)

[9] ddI [10] SQV (Saquinavir)

[11] NVP (Nevirapine) [12] EFV (Efavirenz)

[13] others……………………….

Remark...

Date of stopping or changing regimen…………..…reason……………………………………………

• Date for 3rd time of ARV prescription…………………………..……..………………

Start from [ ] hospital…………… [ ] PHPT………………... [ ] others, specify………………

CD4 result before being prescribed ARV…………..%...............cells, Lab date …/……/……….

ARV regimen [1] GPO virs 30, 40 (3TC+d4T+NVP) [2] GPO virZ 250 (3TC+AZT+NVP)

[3] 3TC (Lamivudine) [4] Combid (3TC+AZT)

[5] AZT (Zidovudine) [6] IDV (Indinavir / Crixivan)

[7] d4T (Stavudine) [8] RTV (Ritronavir / Norvir)

[9] ddI [10] SQV (Saquinavir)

[11] NVP (Nevirapine) [12] EFV (Efavirenz)

[13] others……………………….

Remark...

Date of stopping or changing regimen…………..…reason…………………………………………

• Date for 4th time of ARV prescription…………………………..……..………………

Start from [ ] hospital…………… [ ] PHPT………………... [ ] others, specify………………

CD4 result before being prescribed ARV…………..%...............cells, Lab date …/……/……….

ARV regimen [1] GPO virs 30, 40 (3TC+d4T+NVP) [2] GPO virZ 250 (3TC+AZT+NVP)

[3] 3TC (Lamivudine) [4] Combid (3TC+AZT)

[5] AZT (Zidovudine) [6] IDV (Indinavir / Crixivan)

[7] d4T (Stavudine) [8] RTV (Ritronavir / Norvir)

[9] ddI [10] SQV (Saquinavir)

[11] NVP (Nevirapine) [12] EFV (Efavirenz)

[13] others……………………….

Remark...

Date of stopping or changing regimen…………..…reason…………………………………………

Δ Side effects during when started taking ARV

[ ] No

[ ] Yes, specify the symptoms…………………………………………for …………….days

Solution………………………………………………………………………………….

Δ Long term side effects of the ARV

[ ] No

[ ] Yes, specify ( )

Δ Please compare your health condition before and after taking ARV

[ ] Stronger [ ] weaker [ ] the same

Part 3. General information

1. Marital status [ ] 1. Single [ ] 2. Married [ ] 3. Divorced/ Separated

[ ] 4. Widow/Widower [ ] 5. Others, specify……………………………….

"In case of being married please provide more information, how many time have you been married?...................times. Please give the details:

1st spouse HIV status ( ) 1. HIV-positive ( ) 2. HIV-negative ( ) 3 . Unknown

[1] surviving ☐ separated ☐ living together [2] Died, when……………

2nd spouse HIV status ( ) 1. HIV-positive ( ) 2. HIV-negative ( ) 3 . Unknown

[1] surviving ☐ separated ☐ living together [2] Died, when……………

3rd spouse HIV status ( ) 1. HIV-positive ( ) 2. HIV-negative ( ) 3 . Unknown

[1] surviving ☐ separated ☐ living together [2] Died, when……………

4th spouse HIV status ( ) 1. HIV-positive ( ) 2. HIV-negative ( ) 3 . Unknown

[1] surviving ☐ separated ☐ living together [2] Died, when……………

5th spouse HIV status ( ) 1. HIV-positive ( ) 2. HIV-negative ( ) 3 . Unknown

[1] surviving ☐ separated ☐ living together [2] Died, when……………

2. Ethnicity

[ ] 1. Thai [ ] 2. Chinese [ ] 3. Burmese [ ] 4. Laos

[ ] 5. Hill tribe, specify tribe……………. [ ] 6. Others, specify……………….

3. Religion

[ ] 1. Buddhism [ ] 2. Christianity [ ] 3. Islam [ ] 4. Others, specify……………….

4. Education

[1] no education

[2] still studying, specify …………………………….……………………….…

[3] graduated (specify the educational level) …………………………….…….

5. The number of your family members including you……………

Please specify the relationships……………………………………………………………………..

6. Within last month, did you work?

[ ] 1. Yes, specify your main career

( ) Agriculture, specify…………………….. ( ) Labor, specify………………………….

( ) Merchandising ( ) Employee of a company

( ) Government officer, specify……………. ( ) Student

( ) Housewife/ Househusband ( ) Others, specify………………………..

[ ] 2. No, because ( ) Quit the job for fear that your colleagues would know your HIV testing result.

( ) Forced to resign from your work after your employer knew your HIV testing result.

( ) Still feeling stress after knowing the HIV testing result

( ) Could not work because of the weakness

( ) Have no work to do/ have never done any jobs

( ) Be afraid to be weaker from the work

( ) Others, specify…………………………………………………………

7. Average monthly income………………………THB Remark about the income………………….

8. Have you ever obtained the assistance from the Department of Public Welfare?

[ ] 1. No

[ ] 2. Yes, 500 THB/month allowance on date……./……./…………….

[ ] 3. Yes, 5000 THB complementary income on date……./……./…………….

[ ] 4. Others, specify……………………………………………………………………….

9. Do you have your husband, wife, or relatives being a member of the DCC cohort study?

[ ] 1. No

[ ] 2. Yes, DCC No. ………………..…..

The relationship with you as………………………………………..Tel. No. ………………………

10. Please specify the name of a person of contact, for example, your father, mother, husband/wife, relatives

Name ………………………………………………..…

The relationship with you as………………………………………..Tel. No. ………………………

11. The name of your close friend, who is a Day care center's member and knows where your home is

……………………………………………………. …….Tel. No. ………………………

12. What public health center are you under?..

Part 4. Tuberculin Skin Test (TST)

1. Have you ever been tested for TB infection with TST?

[1] No

[2] Yes, Date of TST……………………. Date of induration size measurement………..……

Induration size…………..mm. Erythema…………………mm.

2. Will the TST must be done for this time?

[1] No, because

[ ] A. Not

[ ] B. Have TB disease for ……… months (specify the treatment start date……..

[ ] C. Could not come to be measure the induration size

[ ] D. Others ……………………………………………….

[2] Yes

Remark: TST will be omitted in case the participant has TB disease and the duration after complete TB treatment is less than 2 years

3. For this time, TST date………/……../………… TST time………………….

Area of TST forearm ☐ Left ☐ Right

Name of the nurse performing TST…………………………………………………

4. Induration size measurement date……/………/…………… Time………………………………

Circumference of the induration perpendicular to the forearm	Circumference of the erythema perpendicular to the forearm
PPD test = …………………………. mm.	PPD test = …………………………. mm.
Measured by…………………………….	

5. The complication from TST

5.1 Blister [1] Yes [2] No

Others (please specify)……………………………………………………………………….......

Part 5. TB Screening

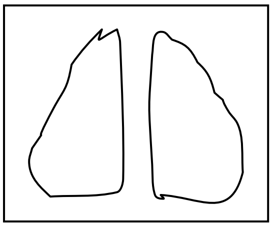

1.Chest X-ray result (to be recorded by physician reading film, check all apply)

[1] Normal [2] Upper Lobe Infiltrate (s)

[3] Infiltrate Not in Upper Lobe(s) [4] Diffuse Infiltrate or interstitial Pattern

[5] Pleural Effusion [6] Mediastinal Lymphadenopathy

[7] Cavitary Lesion [8] Mass or Coin Lesion (not cavity)

[9] Other findings (hyperinflation, rib fracture, etc. Please specify)……………………………………..

CXR is requested on (DD/MM/YYYY)……../………./……….. X-ray film number…………………...

2. Sputum AFB smear results LSN………………… TB STATUS………… TB SUM……………

1st Day sputum………………. Type of sputum [1] Collected [2] Spot

Result: [1] 1+ [2] 2+ [3] 3+ [4] Not seen [5] Not done

2nd Day sputum………………. Type of sputum [1] Collected [2] Spot

Result: [1] 1+ [2] 2+ [3] 3+ [4] Not seen [5] Not done

3rd Day sputum………………. Type of sputum [1] Collected [2] Spot

Result: [1] 1+ [2] 2+ [3] 3+ [4] Not seen [5] Not done

3. Sputum for TB culture: [1] Not done [2] Done, sent date…

Reason for sending for TB culture: [ ] Abnormal CXR result [ ] Chronic cough

Culture LSN	Culture date	Solid result	Solid read	Liquid result	Liquid read	Remark

In case of normal screening result, the participant knows the result from …………... (research assistance)

<u>Summarize from physician</u> (Abnormal TB screening result):

Appointment date…………………………….. Diagnosis: ………………………………………………

Comment: (physical examination result by the doctor)…………………………………………………

……

Treatment…………………………………………………………………………………………………

………..

………..

………..

Part 6. Complete blood count (CBC) and CD4 count

1. CBC (Blood date.......................................Blood time......................CBC time…………….)

   WBC count (5000 - 10000)…………………………………………….........cell/cu.mm

   Hgb (10 - 15)……………………………………………………………........gm/dl

   Hct (40 - 50)……………………………………………………………..…...%

   MCV (80 - 100)……………………………………………………………fl

   MCH (27 - 31)……………………………………………………...………pg

   MCHC (31 - 36)…………………………………………………………g/dl

   RDW (11.2 – 14.8)…..…………………………………………..………%

   RBC (4 - 6)…..…………………………………………………………..M/ul

   Neutrophil (55 - 65)……………………………………………..……..…..%

   Lymphocyte (25 - 35)……………………………………......………..%

   Monocyte (2 - 7)……………………..………………………………..………..%

   Eosinophil (1 - 3)…………………...………………………….……........%

   Basophil (0 - 1)………………………………………………….……….………%

   Platelet .……………………………………………………………..……….

   Platelet Count (140000 - 400000)……………………………..………. cell/cu.mm

2. CD4 count (Date.......................................time..................................)

   CD4 ……………………………………………………...........cell/cu.mm

   %CD4 ……………………………………………………….…..…...%

   CD3 ……………………………………………………...........cell/cu.mm

   %CD3 ……………………………………………………….…..…...%

   WBC ………………………………………………………..…..cell/cu.mm

   %Lymphocyte ……………………………………………...........%
